# Purification and Characterization of a Mucin Specific Mycelial Lectin from *Aspergillus gorakhpurensis*: Application for Mitogenic and Antimicrobial Activity

**DOI:** 10.1371/journal.pone.0109265

**Published:** 2014-10-06

**Authors:** Ram Sarup Singh, Hemant Preet Kaur, Jatinder Singh

**Affiliations:** 1 Carbohydrate and Protein Biotechnology Laboratory, Department of Biotechnology, Punjabi University, Patiala, Punjab, India; 2 Department of Molecular Biology and Biochemistry, Guru Nanak Dev University, Amritsar, Punjab, India; Bangor University, United Kingdom

## Abstract

**Background:**

Lectins are carbohydrate binding proteins or glycoproteins that bind reversibly to specific carbohydrates present on the apposing cells, which are responsible for their ability to agglutinate red blood cells, lymphocytes, fibroblasts, etc. Interest in lectins has been intensified due to their carbohydrate specificity as they can be valuable reagents for the investigation of cell surface sugars, purification and characterization of glycoproteins. The present study reports the purification, characterization and evaluation of mitogenic and antimicrobial potential of a mycelial lectin from *Aspergillus gorakhpurensis*.

**Methods:**

Affinity chromatography on mucin-sepharose column was carried out for purification of *Aspergillus gorakhpurensis* lectin. The lectin was characterized for physico-chemical parameters. Mitogenic potential of the lectin was evaluated against splenocytes of Swiss albino mice by MTT assay. Antimicrobial activity of the purified lectin has also been evaluated by disc diffusion assay.

**Results:**

Single-step affinity purification resulted in 18.6-fold purification of the mycelial lectin. The molecular mass of the lectin was found to be 70 kDa and it was composed of two subunits of 34.8 kDa as determined by gel filtration chromatography, SDS-PAGE and MALDI-TOF analysis. pH optima of the lectin was found to be 6.5–9.5, while optimum temperature for lectin activity was 20–30°C. Lectin was stable within a pH range of 7.0–10.5 and showed fair thermostability. EDTA did not affect lectin activity whereas it was found susceptible to the denaturants tested. MTT assay revealed strong mitogenic potential of *A. gorakhpurensis* lectin at a concentration upto 150 µg/mL. Antimicrobial activity assay showed its potent antibacterial activity against *Bacillus cereus*, *Staphylococcous aureus* and *Escherichia coli* and marginal antifungal activity against *Saccharomyces cerevisiae.*

**Conclusion:**

This is the first report on the mitogenic and antimicrobial potential of *Aspergillus gorakhpurensis* lectin. The results will provide useful guidelines for further research in clinical applications of this lectin.

## Introduction

Lectins are proteins or glycoproteins of non-immune origin found in a diversity of organisms. They have at least one non-catalytic domain that exhibits reversible binding to specific monosaccharides or oligosaccharides. They can bind to the carbohydrate moieties on the surface of erythrocytes and agglutinate the erythrocytes without altering the properties of carbohydrates [1]. The specificity and strength with which the carbohydrate recognition domain binds to saccharide is the key element of their biological role [2]. Lectins are present in all kinds of organisms from viruses to man, but were initially identified in the plant kingdom due to their hemagglutinating activity [3]. Biological activity of lectins is potentially useful in biotechnological and biomedical applications [4]. Carbohydrate binding properties have been applied in the fields of immunology, cell biology, cancer research and genetic engineering [5]. Extensive literature is available on the activity of lectins in a number of different tissues and processes, their role in cell biology and as potential therapeutic agent especially for cancer, molecular/cell biology and immunology. Lectins have been used to fractionate animal cells including B and T lymphocytes and also illustrate changes in cell surface architecture following virus or parasite infection [6]. Lectins interact with cells and show mitogenicity i.e., the triggering of quiescent, non-dividing lymphocytes into a state of growth and proliferation [7]. Over the last few decades, lectins have been investigated owing to their variety of interesting biological properties including antibacterial, antifungal, insecticidal and antiviral activities [8].

An increasing number of lectins from plants and animals have been purified and characterized, however the information on lectins isolated from fungal sources remains limited. Fungal lectins are drawing greater attention as many of them exhibit interesting physiological effects such as lymphomitogenic activity, immunomodulatory effect, suppression of cell proliferation and antitumor activity [9]. There are many reports on lectins from microfungi, but their physiological role remains uncertain. Various fungal lectins have been purified by affinity chromatographic approach, employing lectin sugar interaction, ion-exchange, gel filtration or hydrophobic interaction chromatographies singly or in combinations. In many instances, at least one of these chromatographic columns has also been applied with affinity chromatographic approach to attain the desired purity level. [10].

In an attempt to detect the occurrence of lectins in *Aspergillus* species, our group in a preliminary study has screened 16 species of Aspergilli. Five of them, *A. acristatus, A. carbonarius, A. panamensis, A. gorakhpurensis,* and *A. fischeri* were found to possess lectin activity [11]. The present work was aimed to purify the lectin from *Aspergillus gorakhpurensis* using a simple and most effective technique. The purified lectin was characterized and its mitogenic as well as antimicrobial potential was determined.

## Materials and Methods

### Fungal growth and maintenance

*Aspergillus gorakhpurensis* (MTCC 547) was procured from Microbial Type Culture Collection (MTCC), Institute of Microbial Technology, Chandigarh, India. The culture was grown on Czapek malt agar slants containing (in g/L): sodium nitrate 3.0, potassium chloride 0.5, magnesium sulphate 0.5, ferrous sulphate 0.01, dipotassium hydrogen orthophosphate 20.0, sucrose 30, malt extract 40 and agar 30. The strain was cultivated by inoculating 5 agar discs (10 mm diameter) covered with mycelia into Czapek malt liquid medium (500 mL) in Erlenmyer flasks (1L) followed by incubation under stationary conditions at 30°C for 7 days.

### Extraction of intracellular lectin

The mycelium was recovered from the culture broth by filtration and thoroughly washed using phosphate buffered saline (PBS; 0.1 M; pH 7.2). It was dried by pressing between folds of filter paper. Intracellular lectin was extracted in phosphate buffered saline (0.1 M; pH 7.2) supplemented with 1 mM benzamidine. Fungal mycelium was homogenized using high speed homogenizer (Ultra-Turrax T25 basic, IKA-Werke) and ground using a pestle and mortar as described earlier [12]. The extract was centrifuged (3000×g; 20 min; 4°C) and supernatant was assayed for lectin activity.

### Purification of *A. gorakhpurensis* lectin

Lectin was purified using affinity chromatography. Mucin from porcine stomach (Sigma) was coupled to CNBr-activated Sepharose-4B (GE Healthcare, USA) according to manufacturer's instructions. The affinity matrix was packed into a glass column (1×10 cm, GE Healthcare, USA) and equilibrated with phosphate buffered saline (0.1 M; pH 7.2). Crude lectin extract was loaded at reduced flow rate. The column was then washed with the same buffer till A_280_ of the fraction (1.0 mL) dropped below 0.02. The bound lectin was eluted by 0.02 M EDTA at a reduced flow rate of 0.5 mL/min. The eluted fractions were monitored for lectin activity and protein content [13]. Lectin positive fractions were pooled and dialysed extensively against phosphate buffered saline (0.1 M; pH 7.2) using Snake Skin Dialysis tubing (MWCO 10 kDa; Pierce Biotech, USA). Lectin titre and protein content of the combined fractions was analyzed. The lectin positive fractions were concentrated in ultraspin unit “Centricon” (30 kDa; Millipore, USA). Lectin titre and protein content of the concentrated fractions was determined.

### Erythrocyte preparation and hemagglutination assay

Human blood type O erythrocytes were used for determination of hemagglutination activity as described previously [11]. Erythrocyte cells were adjusted to 2% suspension. Lectin preparation (20 µL) was serially diluted with phosphate buffered saline (0.1 M; pH 7.2) in a microtitre U bottom plate with 96 wells followed by incubation with 20 µL of 2% suspension of human blood type O erythrocytes at room temperature for 1 h and then allowed to settle at 4°C. Lectin activity was expressed as hemagglutination titre which is defined as the reciprocal of the end point dilution that achieved hemagglutination.

## Characterization of Purified Lectin

### Determination of molecular weight

The molecular weight of the purified lectin was estimated by SDS-PAGE (10%) under denaturing conditions according to Laemmli [14]. Dissociation and reduction of the protein was performed by heating it for 5 min at 100°C with 0.1% SDS in the presence of 2-mercaptoethanol (0.1%). The gel was run on a Mini-Protein III electrophoretic system (Bio-Rad Laboratories Inc., USA) at a constant voltage (50 V) and protein bands were visualised by staining with 0.05% Coomassie Brilliant Blue R250. Molecular mass was estimated by comparing the electrophoretic mobility of the sample against molecular mass marker proteins (7.0–201.2 kDa; Bio-Rad Laboratories Inc., USA). Molecular mass of the purified lectin was confirmed by gel exclusion chromatography using Sephadex G-100 (GE Healthcare, USA) packed into a glass column (1×30 cm; GE Healthcare, USA). The column was pre-equilibriated using phosphate buffered saline (0.1 M; pH 7.2). Aprotinin (6.5 kDa), Ribonuclease A (13.7 kDa), Carbonic anhydrase (29 kDa), Ovalbumin (44 kDa) and Conalbumin (75 kDa) from GE Healthcare, USA were used as the reference proteins. Molecular mass of the purified lectin was also determined by matrix-assisted laser desorption ionization time-of-flight (MALDI-ToF) on an Autoflex (Bruker Daltonics, Billerica, USA). The spectrum was acquired in a linear mode at a detector voltage of 1.65 kV. Sinapinic acid was used as the matrix.

### Carbohydrate specificity of purified lectin

Hemagglutination-inhibition tests were performed to characterize the purified lectin for its carbohydrate specificity as described previously [11]. All the simple sugars, complex sugars and glycoproteins used earlier to determine the carbohydrate specificity of the crude fungal extract were tested for purified lectin.

### pH dependence and thermal inactivation

To determine the optimal pH for lectin activity, hemagglutination assay was carried out using buffers of pH 5.0–10.0 at room temperature. pH stability was determined by incubating purified lectin (50 µL) with buffers (450 µL) at pH 1.5–12.5 in different aliquots at 4°C. Samples were neutralized and activity was assayed at 0 h, 2 h, 4 h and 24 h to elucidate the stability of lectin at varied pH. The buffers used were 0.1 M glycine-HCl buffer (pH 1.5–3.5), 0.1 M sodium acetate-acetic acid buffer (pH 4.0–5.0), 0.1 M phosphate buffer (pH 5.5–6.0), 0.1 M Tris-HCl buffer (pH 6.5–8.0) and 0.1 M glycine-NaOH buffer (pH 9.0–12.5). To determine the optimal temperature for lectin activity, agglutination assay was carried at 4, 20, 25, 30, 35 and 40°C. Thermal stability of lectin was assessed by incubating the purified lectin over a temperature range of 25–100°C with increments of 5°C in a water bath. Samples were drawn after 10, 20 and 30 min of incubation, chilled in ice bath and assayed for hemagglutination. Lectin activity at any given temperature was compared to control samples incubated at 4°C and expressed as percentage relative activity.

### Stability analysis with denaturants and EDTA

Purified lectin was incubated at 4°C with an equal volume of urea, thiourea and guanidine-HCl in phosphate buffered saline (0.1 M; pH 7.2) at a final concentration of 1–4 M. Control samples were incubated with an equal volume of phosphate buffered saline (0.1 M; pH 7.2) at the same temperature. Agglutination assay was carried out at 0 h, 2 h, 4 h and 24 h. Activity at any concentration at a given time was expressed as percentage relative activity compared to control. To determine the effect of EDTA, the purified lectin was incubated at 4°C with an equal volume of EDTA (0.2 M) for 24 h. Control samples were incubated with an equal volume of phosphate buffered saline (0.1 M; pH 7.2) at the same temperature. Agglutination assay was carried out at 0 h, 2 h, 4 h and 24 h. Activity at any concentration at a given time was expressed as percentage relative activity as compared to control.

### Effect of metal ions on lectin activity

The effect of divalent metal ions on lectin activity was assessed by extensive dialysis of purified lectin against 10 mM EDTA for 48 h at 4°C and then against deionized water. The haemagglutination activity was tested before and after addition of 40 mM Ca^+2^, Mn^+2^, Mg^+2^ and Fe^+2^ ions.

### Carbohydrate analysis

Carbohydrate content of the purified lectin was estimated by anthrone reagent method using D-glucose as standard [15].

## Evaluation of Mitogenic Potential of Purified Lectin

### Animals

Male Swiss albino mice (20–25 g) were procured from Central Research Institute, Kasauli (H.P.), housed under controlled temperature (25±2°C) with 12 h light/dark cycle and maintained on standard pellet diet (Kissan Feeds Ltd., Mumbai, India). Mice were sacrificed by cervical dislocation and their spleen was excised aseptically.

### Ethics statement

The experimental protocol was approved by Institutional Animals Ethics Committee, Punjabi University, Patiala (Permit No. 107/99/CPCSEA/2013/35) and care of the animals was carried out as per the guidelines of Committee for the purpose of Control and Supervision of Experiments on Animals (CPCSEA), Ministry of Environment and Forests, Government of India.

### Cell preparations

Isolated spleen was perfused in sterilised RPMI 1640 medium containing heat inactivated fetal bovine serum (10%), streptomycin (5 mg/mL) and penicillin (5000 U/mL). Viable cell count was adjusted to 2×10^6^ cells/mL in RPMI 1640. Viable cells were counted microscopically on a haemocytometer.

### Assay of mitogenic potential

Mitogenic potential of purified lectin of *A. gorakhpurensis* was determined against splenocytes as described by Xiong et al. [16]. A stock solution of purified lectin (1 mg/mL) was prepared in phosphate buffered saline (0.1 M; pH 7.2) and sterilised by passing through 0.22 µm syringe filter (Millipore, USA). RPMI 1640 medium supplemented with L-glutamine (1 mmoL/L), streptomycin (100 µg/mL), penicillin (100 U/mL), heat-inactivated fetal bovine serum (10%) and Hepes (25 mM) was adjusted to pH 7.2 with sodium bicarbonate and filter sterilized. Lectin stock solution was diluted to working concentrations (1, 5, 10, 50, 100, 150, 200, 250 µg/mL) in RPMI 1640 medium. Splenocyte suspension (100 µL) was added in each well of flat bottom 96-well microtitre plate (Tarsons Products Pvt. Ltd., India). Different concentrations of lectin (100 µL) were added to different wells and incubated at 37°C in a humidified atmosphere containing 5% CO_2_ for 72 h. Cells supplemented with Concanavalin A (Con A 5 µg/ml) were taken as positive control and cells without the addition of purified lectin were taken as negative control. Contents of each well were centrifuged (400×g, 10 min, 4°C) and supernatant was discarded. MTT reagent (100 µL) was added to cell pellets and incubated for 4 h in 5% CO_2_ atmosphere at 37°C. MTT formazan crystals formed were dissolved completely in 100 µL dimethyl sulphoxide (DMSO) and plates were read on a Microplate reader (Biorad Laboratories Inc., USA) at 570 nm/630 nm. The experiments were carried out in triplicates and results were expressed as Mean ± SD.

## Antimicrobial Activity Determination

Antibacterial and antifungal activities of the purified *A. gorakhpurensis* lectin were evaluated as described by Chumkhunthod et al. [17]. For the antibacterial assay, *Bacillus cereus* MTCC 1270, *Staphylococcus aureus* MTCC 6908, *Pseudomonas aeruginosa* MTCC 1034 and *Escherichia coli* MTCC 42 were used. These strains were inoculated in nutrient broth and grown overnight under agitation at 37°C. Mc Farland suspension (0.5) was prepared using sterile phosphate buffered saline (0.1 M, pH 7.2). Muller Hinton agar plates were inoculated by three-dimensional swab technique. Sterile filter paper discs were impregnated with 20 µL of purified lectin (specific activity 40.00 titre/mg) and then placed on the inoculated agar plates and incubated at 37°C for 24 h. Antibiotic ampicillin (10 mg/mL) impregnated on filter paper discs was used as a positive control. The plates were examined for zone of inhibition that indicates the degree of susceptibility of the test organism.

Agar diffusion method was used for antifungal activity assay. *Saccharomyces cerevisiae* MTCC 170, *Candida albicans* MTCC 183 and *Aspergillus niger* MTCC 281 were grown in 2% (w/v) malt extract broth (MEB) for 48 h at 30°C. Fungal suspensions having concentration 10^7^ **spores/mL were prepared and then streaked on 2% (w/v) malt extract agar plates. The purified lectin was applied as described above. One hundred units of nystatin impregnated on filter paper discs were used as positive control. Inhibitory zones were detected after incubation for 72 h at 30°C. All the tests were performed in triplicates.

## Results and Discussion

In a preliminary investigation on the screening of *Aspergillus* species for occurrence of lectins, lectin from *A. gorakhpurensis* was found to be a panagglutinin [11]. It exhibited slight preference for N-acetyl-D-galactosamine containing blood type A erythrocytes over B and O. Porcine stomach mucin was found to be the most potent inhibitor of *A. gorakhpurensis* lectin mediated hemagglutination besides having a complex carbohydrate specificity towards N-acetyl-D-galactosamine, chondroitin-6-sulphate, fetuin, N-glycolyl neuraminic acid, D-mannitol and D-trehalose dihydrate. The lectin was purified from its crude extract according to its carbohydrate binding specificity. Carbohydrate affinity chromatography is a particular affinity chromatography technique in which carbohydrate adsorbants are used for purification of glycan binding proteins or lectins. It is a simple one step purification method in which advantages of the specific properties of the lectin are exploited [18]. In this manuscript, authors report the successful application of this technique on cyanogen bromide activated sepharose 4B derivatized with porcine stomach mucin for purification of *A gorakhpurensis* lectin. The purified lectin has been characterized and was found to possess a remarkable mitogenic activity towards mouse splenocytes. It was also found to exhibit potent antimicrobial activity. It is the first report on the purification of *A. gorakhpurensis* lectin having mitogenic and antimicrobial activity.

### Affinity purification of *A. gorakhpurensis* lectin

**Figure 1:**
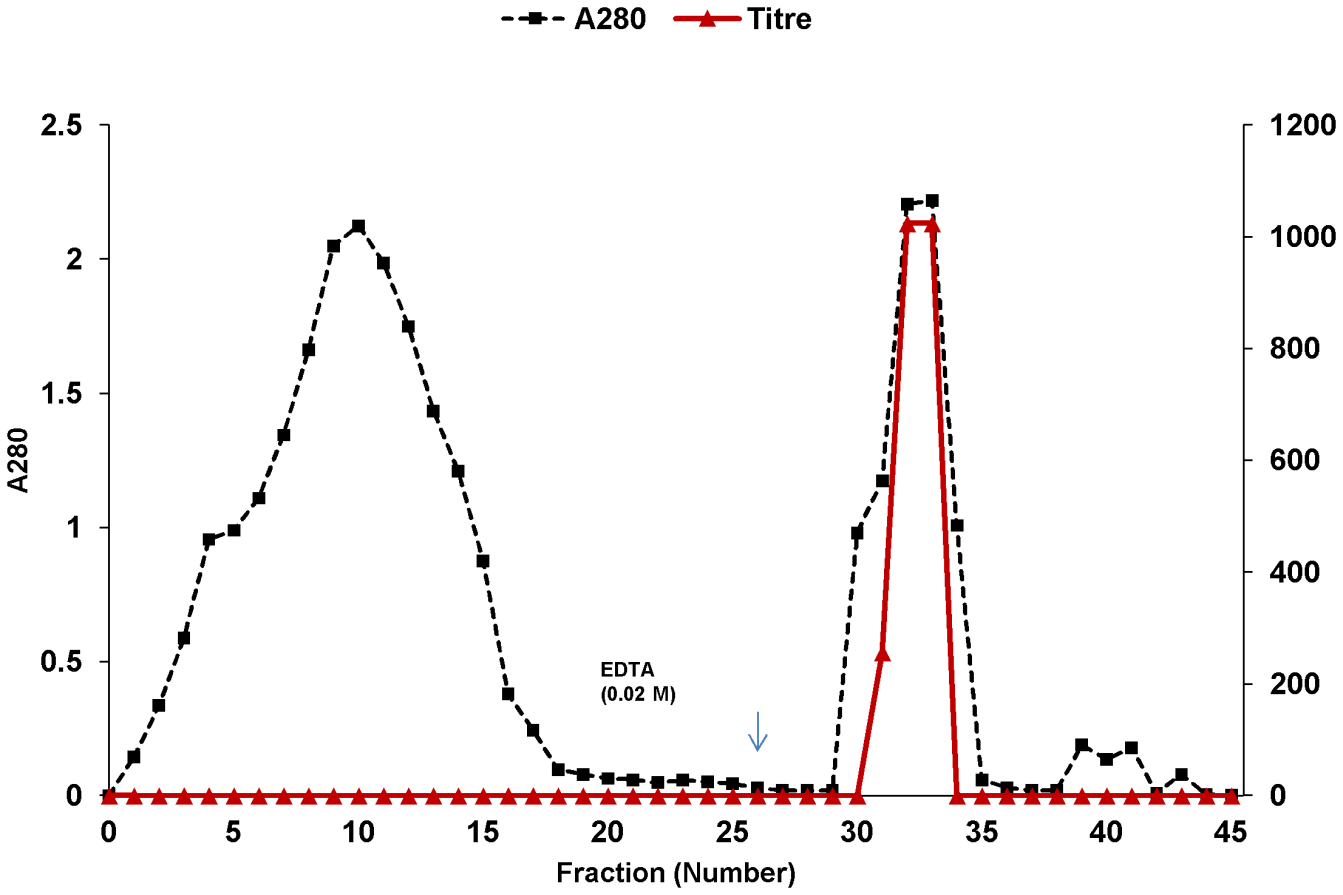
Affinity purification of *A. gorakhpurensis* lectin on mucin-Sepharose 4B column. Fractions (1.0 mL) were collected and analyzed for protein content and titre.

**Figure 2:**
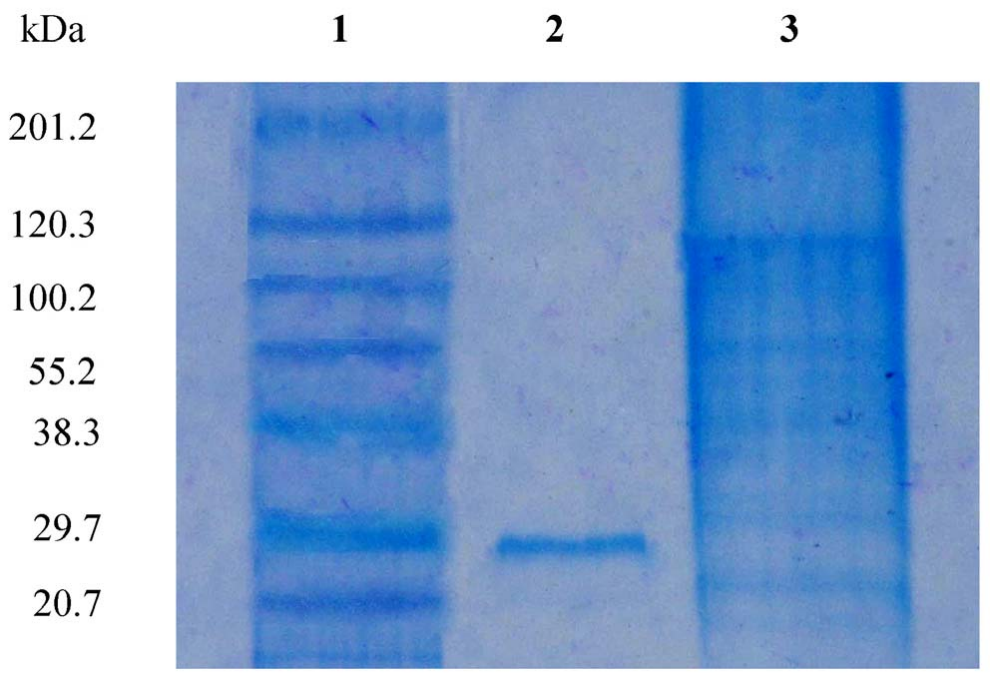
Sodium Dodecyl Sulphate-Polyacrylamide Gel of *A. gorakhpurensis* lectin. Lane 1: Molecular weight markers from top: myosin (201.2 kDa); β-galactosidase (120.3 kDa); bovine serum albumin (100.2 kDa); ovalbumin (55.9 kDa); carbonic anhydrase (38.3 kDa); soyabean trypsin inhibitor (29.7 kDa) and lysozyme (20.7 kDa), Lane 2: Porcine stomach mucin-Sepharose 4B fraction, Lane 3: Crude.

**Figure 3:**
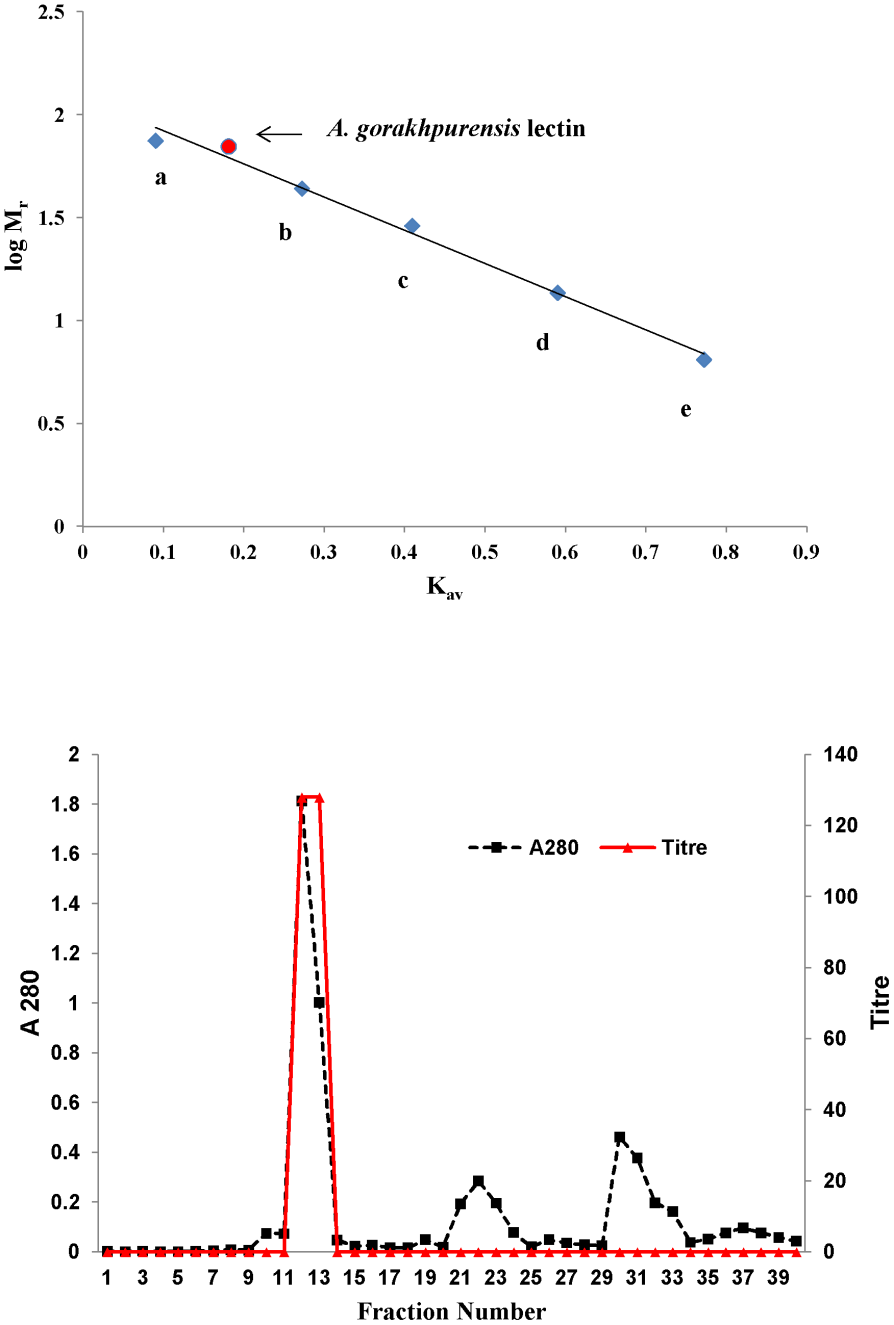
a. Molecular mass determination of purified *A. gorakhpurensis* lectin by gel filtration chromatography on Sephadex G-100 column. (a) Conalbumin 75 kDa, (b) Ovalbumin 44 kDa, (c) Carbonic anhydrase 29 kDa, (d) Ribonuclease A 13.7 kDa, (e) Aprotinin 6.5 kDa. b. Elution profile of *A. gorakhpurensis* lectin on Sephadex G-100 column.

**Figure 4:**
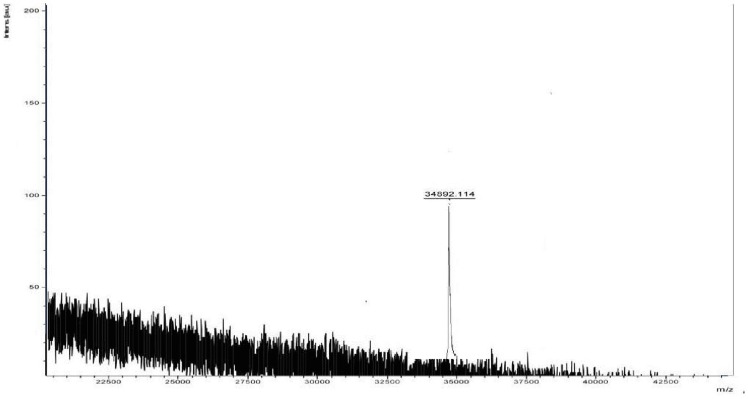
Molecular mass determination of *A. gorakhpurensis* lectin by MALDI-TOF.

*A. gorakhpurensis* lectin was eluted in two fractions (fraction No. 32 and 33) and the titre of the combined fractions was 256 with a specific activity of 36.46 titre/mg (**Table 1**). The elution profile of *A. gorakhpurensis* lectin on mucin-sepharose coloumn is depicted in **Figure 1**. Affinity purification yielded 50% activity with 18.6-fold purification. Lectin from *Aspergillus terricola* has been efficiently purified in a single step on mucin-sepharose column [19]. Affinity chromatography has been employed to purify lectins from *Sclerotinia sclerotiorum* and *Arthobotrys oligospora* [20], [21]. Different affinity gels were attempted for the purification of *Aspergillus nidulans* lectin but elution could not be achieved. The lectin was purified in a multistep process using conventional approaches [22]. A 18-fold purification of *A. gorakhpurensis* lectin obtained by single step was in the same range as reported for other purified fungal lectins by multistep conventional chromatographic techniques [23], [24]. Low concentration of chelating agent EDTA has been previously used to elute lectins from affinity matrices [18]. SDS-PAGE analysis of purified lectin yielded a single band with apparent molecular mass of 30.0 kDa (**Figure 2**), demonstrating the purity of the final product. The active fractions obtained from affinity column were concentrated by ultrafiltration before applying to Sephadex G-100 column for molecular weight estimation. Gel filtration of the protein resulted in a single peak at an elution volume corresponding to molecular mass of 70 kDa (**Figure 3a, b**). The MALDI-TOF spectrum shown a main peak at *m/z* 34892 (**Figure 4**). The results of SDS-PAGE, gel filtration and MALDI-TOF mass analysis indicate the molecular mass of *A. gorakhpurensis* lectin is 70 kDa and it is a homodimer of 35 kDa subunits. Molecular mass of most fungal lectins ranges from 15-90 kDa. Generally most of them are dimeric proteins and subunits are held together by non-covalent interactions with a few exceptions [25]. A lectin has been purified from the mycelia of *Punctularia atopurpurascens* using affinity chromatography on chitosan-sepharose. SDS-PAGE of the lectin proved the presence of one band with a molecular mass of 67 kDa [26]. The apparent molecular mass of purified lectins from *Aspergillus* species has been reported in the range of 32–35 kDa [22], [27], [28]. *A. gorakhpurensis* lectin was found to be a glycoprotein with carbohydrate content of 9.0%. *Aspergillus nidulans* lectin has also been reported to be a glycoprotein with carbohydrate content of 2.54%. [22]. Lectins from *Sclerotinia sclerotiorum* and *Rhizopus stolonifer* are reported to lack carbohydrate domains whereas, a high proportion of carbohydrates is present in *Macrophomina phaseolina* and *Aspergillus terricola* lectins amounting to about 10.4%, and 9.76%, respectively [10].

**Table 1:** Summary of purification of *A. gorakhpurensis* lectin.

Step	Volume (mL)	Total Titre	Total Protein (mg)	Specific Activity (Titre/mg)	Yield (%)	Fold Purification
Crude extract	4.0	128	65.64	1.95	100	1
Affinity purified lectin	2.0	256	7.02	36.46	50	18.6
Ultrafiltrate	1.0	256	6.40	40.00	50	20.5

The lectin was purified by affinity chromatography on mucin-sepharose column and fractions (1.0 mL) were collected. Bound lectin, eluted by EDTA (0.02 M) was recovered in only 2 fractions. Combined fractions were dialysed and assayed for lectin activity and protein content.

### Carbohydrate specificity of purified lectin

The inhibitory potentials of the saccharides were found to correspond to the results of our earlier findings on crude fungal extract [11]. The purified lectin exhibited a very strong affinity for porcine stomach mucin. Chondroitin-6-sulphate was also found to be a strong inhibitor and out of the various polysaccharides, pullulan, dextran and starch inhibited the lectin activity.

### pH dependence and thermal inactivation

Purified lectin exhibited maximum lectin activity (128) at pH 6.5–9.5. Lectin activity was completely stable between pH 7.0–10.5 after 24 h, while above and below this range, a substantial loss in activity was observed. Optimal lectin activity was observed at 20–30°C. The lectin retained 100% activity upto 60°C even after 30 min of incubation. Lectin activity was completely lost at higher temperatures. Fungal lectins show wide disparity in pH and thermal stability profiles. Lectin isolated from mushroom *Ganoderma capense* showed complete retention of lectin activity at 100°C for 1 h and was also stable within pH range of 3.0–12.0 [29]. Lectin stability at elevated temperatures could be attributed to the presence of high carbohydrate content [10]. Lectin from *Aspergillus nidulans* completely decayed at temperature higher than 40°C [21]. *Aspergillus terricola, Lentinus squarrosulus* and *Rhizopus stolonifer* lectins have also been reported to exhibit good thermostability [19], [30], [31].

### Stability analysis with EDTA and denaturants

*A. gorakhpurensis* lectin was stable after 24 h incubation in EDTA. Lectin was denatured upon incubation with urea, thiourea and guanidine HCl. Lectin activity was not affected in 1 M urea, while 75% and 93.75% loss of activity was observed in 2 M and 3 M urea, respectively after 24 h. Lectin retained 12.5% and 6.25% activity in 1 M thiourea and guanidine HCl, respectively after 24 h. The lectin completely degraded in 4 M urea, 2–4 M thiourea and 2–4 M guanidine HCl. Denaturation by urea, thiourea and guanidine-HCl indicates the globular nature of lectin which is stabilized mainly by hydrophobic interactions [26]. Stability of lectin with EDTA and its denaturation by urea, thiourea and guanidine-HCl is in agreement with earlier reports of lectins from *Aspergillus terricola* and *Aspergillus nidulans* [19], [22].

### Effect of metal ions on lectin activity

Lectin activity remained unaltered after metal ion chelation with EDTA or in the presence of Ca^+2^, Mn^+2^, Mg^+2^ and Fe^+2^ ions. This observation indicates that lectin does not require metal ions for binding. No effect of EDTA treatment has been reported on lectin activity of *Aspergillus terricola* [19], *Aspergillus nidulans* [22], *Fusarium solani* [32] and *Chlorophyllum molybdites* [33]. Removal of metal ions by EDTA disrupts the carbohydrate binding site and reduces lectin activity of many plant lectins [34].

### Mitogenic potential of *A. gorakhpurensis* lectin

**Figure 5:**
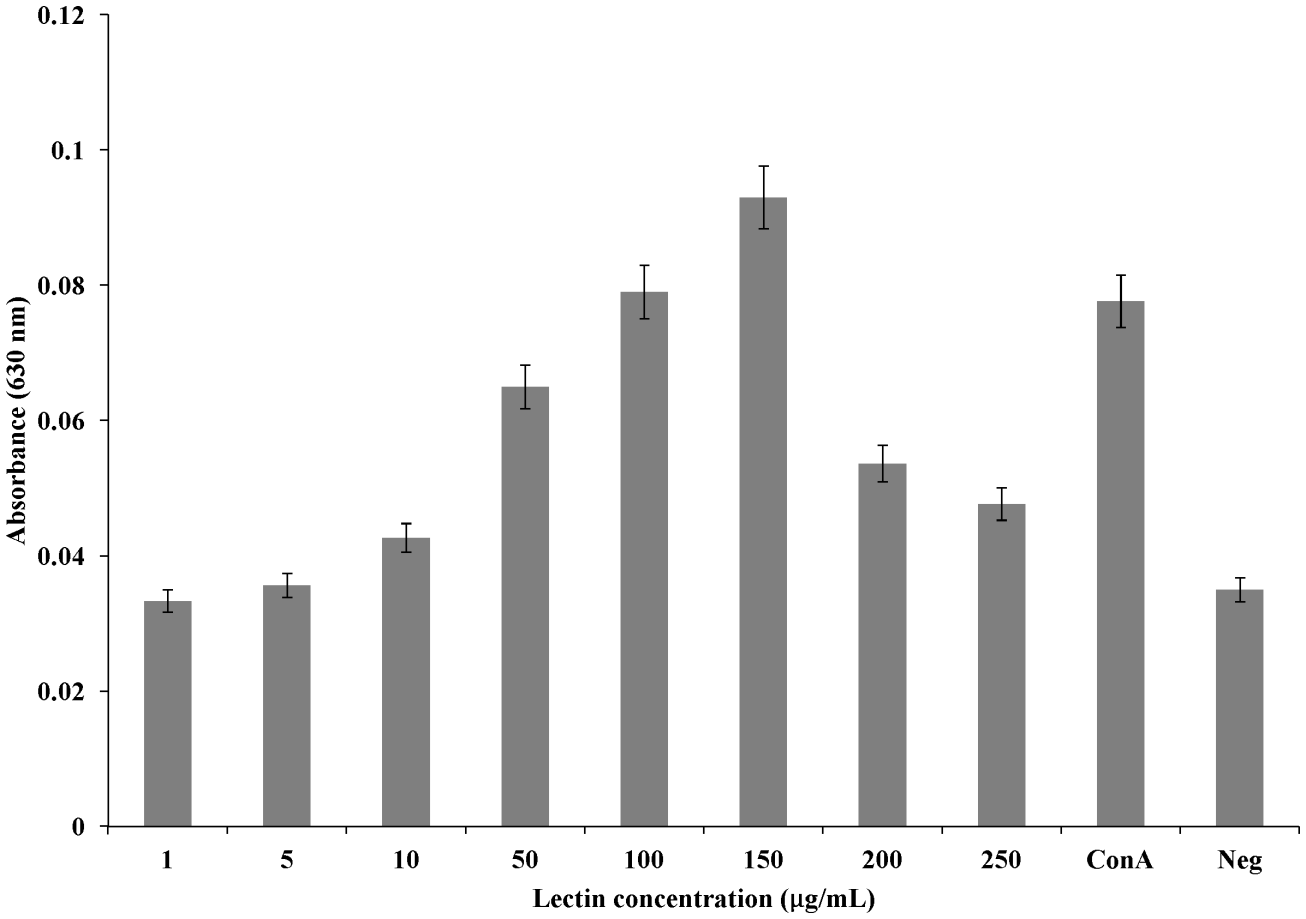
Mitogenic response of mouse splenocytes to purified *A. gorakhpurensis* lectin. Con A, Concanavalin A lectin (Positive control); Neg, Negative control (Cells without the addition of any mitogen). Data is presented as Mean ± SD.

*A. gorakhpurensis* lectin showed mitogenic activity for mouse splenocytes. The degree of activity was identical to that of concanavalin A at a lectin concentration of 100 µg/mL. The activity was found dose dependent, showing optimal concentration of 150 µg/mL (**Figure 5**). At this concentration, the mitogenic activity was greater than the standard concanavalin A. Further increase in lectin dose was accompanied by decrease in viable cell count as evidenced by decrease in absorbance. By far, mitogenic activity of microfungi lectins has only been reported from *Aspergillus nidulans* [22]. Mitogenic lectins are of clinical importance as they can be used in the examination of biochemical events leading to the conversion of resting cells to actively growing ones. Lectins from higher fungi namely *Boletus edulis*, *Cordyceps militaris* and *Pleurotus citrinopileatus* are known to be mitogenic with respect to murine splenocytes. Antimitogenic effect of *Xylaria hypoxylon* lectin on mouse splenocytes has been reported, whereas, lectin from *Pleurotus flabellatus* is non-mitogenic [35].

### Antimicrobial activity

The purified lectin was screened for its antimicrobial activity by the disc diffusion method by measuring the diameter of inhibitory zones (**Table 2**). It showed greater antibacterial activity against Gram positive bacteria as compared to Gram negative bacteria. *Bacillus cereus* showed the maximum zone of inhibition (20±0.25 mm). Marginal antifungal activity was observed against *Saccharomyces cerevisiae* (9±0.25 mm) as compared to the standard antifungal antibiotic used (30±0.012 mm). The growth of *Candida albicans* and *Aspergillus niger* remained unaffected in the assay. The antibacterial activity of conventional antibiotics has been shown to be greater than that of purified *A. gorakhpurensis* lectin. This is the first report on the antimicrobial activity of a purified microfungal lectin. It is interesting to study the antimicrobial activity of lectins because of the abundant prevalence of pathogenic microorganisms in the environment. The interaction of lectins with bacteria is based on cell wall carbohydrates or extracellular glycans. Since all microbes express surface exposed carbohydrates, the carbohydrate binding site plays a key role in interaction of lectins with bacteria. *A. gorakhpurensis* lectin may possibly interact with these carbohydrates and this interaction may promote alterations in the flow of nutrients, thereby explaining the bacteriostatic effect. In contrast to Gram positive bacteria, cell wall of Gram negative bacteria possesses higher lipids. Decreased effect on Gram negative bacteria and absence of antifungal activity may be due to the absence of specific carbohydrates on the surface of cell wall of these organisms or due to the inaccessibility of these carbohydrates to *A. gorakhpurensis* lectin. The major component of fungal cell wall is chitin, which is a polymer of β-(1, 4)-N-acetyl-D-glucosamine [36]. *A. gorakhpurensis* lectin does not exhibit specificity for N-acetyl-D-glucosamine thus corroborating its inability to bind to the fungal cell wall. Crude lectin extracts of *Schizophyllum commune* and *Penicillium* species have been reported to possess some antifungal activities [16], [37]. Purified lectin from *Gymnopilus spectabilis* has been found to produce growth inhibition haloes against *Staphylococcus aureus* and *Aspergillus niger* [38].

**Table 2:** Antimicrobial activity of purified *A. gorakhpurensis* lectin.

Test organism	Zone diameter (mm)
**Bacteria**	
*Bacillus cereus*	20±0.25
*Staphylococcous aureus*	18±0.12
*Pseudomonas aeruginosa*	nil
*Escherichia coli*	10±0.05
Control	30±0.012
**Fungi**	
*Saccharomyces cerevisiae*	9±0.25
*Candida albicans*	nil
*Aspergillus niger*	nil
Control	30±0.012

## Conclusions

Among 1.5 million fungal species, few fungal lectins have been reported so far and very little information is available regarding their functions and biological roles. We herein present the first report on the purification and characterization of a lectin from *A. gorakhpurensis.* The strong mitogenic and antimicrobial properties of this lectin make it a strong candidate for future studies in glycobiology and biomedical applications. None of the purified microfungal lectins reported till date exhibit antimicrobial activity. The current study adds to the low number of lectins purified in a single step and gives an insight to the possible applications of this lectin.
